# Artificial Respiration

**Published:** 1870-01

**Authors:** Sumner Rhoades

**Affiliations:** Of Lowville, N. Y.


### ARTICLE X.

### *Artificial Respiration.*

By Sumner Rhoades, M.D.,

Of Lowville, N. Y.

Marshall Hall directs the patient to be rolled sixteen times a minute alternately on his stomach, and on one side, pressure to the thorax and abdomen to be applied when he is lying on his stomach. Dr. H. R. Silvester places the patient on his back, with the shoulders raised, and the tongue drawn forward. He then alternately lifts the ribs and sternum by extending the patient's arms up by the side of his head, and then compresses the sides of the chest by the patient's arms. Pacini, of Florence, in Italy, has a method, "which consists in placing the patient on his back, on a table or bed, the operator having his abdomen against the head of the patient, placing his hands in the axillæ on the dorsal aspect, and then pulling the shoulders toward him with an upward movement at the same time. The shoulders are then relaxed, then the former movement, and so on alternately." Dr. W. P. Bain (standing, I suppose, at the head of his patient,) "places his fingers in the axillæ in their front aspect, with his thumbs over the outer ends of the clavicles, and draws the shoulders towards him. On relaxing his hold, the shoulders and chest return to their former position, and so on with alternate motion."

The Ready Method, as that of Marshal Hall has been called, was published in April, 1856, and was everywhere hailed with enthusiasm. When Dr. Silvester's method was published in 1858, it suggested to me the one I have ever since practised, and which is as follows: Wherever I find my patient, I lay him on his back, place myself at his head, put my hands under his shoulders, and my fingers in his axillæ; then whenever I myself inspire, I draw and lift his shoulders toward me; and whenever I expire, I let go the shoulders, and with my own hands firmly press the sides of his chest. Meantime I have an assistant open the mouth, and see if the tongue has fallen back from the lower incisor teeth, and if so I have him draw it out, using for the time his thumb and finger. If the process has to be long continued, I presently resign my place to some strong, intelligent bystander, cautioning him not to work too fast, while I look after the use of the galvanic battery, friction of the extremities, and sometimes enemata of capsicum and ammonia. If, when the shoulders are lifted, I see that the chest is not proportionately elevated, I suspect obstruction of the glottis by the tongue. If need be, I use a tenaculum to hold the tongue forward, rather than pinch it between the upper and lower front teeth, as advised by Silvester; for, as the nares are likely to be partially obstructed by frothy fluid, the mouth should be kept open. Once, on a river bank, some distance from any dwelling, having with me no tenaculum, I passed a threaded needle through the tip of the tongue, a lady present supplying me thread by unraveling the hem of her dress. I ought instead to have borrowed a garter, and with a pin made of it an elastic band to pass over the tongue and under the chin. Upon a child born asphyxiated at Ithaca, N. Y., August 4. 1860, I thus kept up artificial respiration twenty-five minutes before there was the slightest effort at a natural inspiration. But I was all the time assured of success by the improvement in the complexion, which showed that my work was effecting oxygenation of the blood. In 1861, I thus unavailingly kept up artificial respiration in a drowned boy, while on a skiff, and in an express wagon, going to his father's house. In August, 1865, after apparent death from amputation of the thigh, chloroform having been given, a patient of mine, by artificial respiration thus practiced, was temporarily resuscitated, so as to speak aloud, intelligently, and repeatedly, to the profoundest astonishment of physicians, and all others there present.

Marshall Hall's method, no doubt, led the way to the more efficient one of Silvester; and that suggested the one of Pacini, and the similar one of mine; while that of Pacini suggested Dr. Bain's. The three last I deem preferable to Silvester's, because of the awkward and unnecessary use he makes of the patients arms, and the difficulty he must occasionally experience from rigidity of the muscles. Pacini's and Bain's methods (my first knowledge of which was obtained a fortnight ago from Dr. Butler's Compendium) are I think, inferior to mine, in that they do not aid the patient in the process of expiration.

I was led to criticise Dr. Silvester's plan, because (1) in reducing dislocation of the hip, I had experienced the advantage of making extension from the femur, rather than from the ankle. (2.) I had observed the relief given to the exhausted, parturient woman, by pressing one's hands firmly on the top of her shoulder during the last pains of labor. (3.) I like directness of purpose, and hate all roundabout ways of doing things.

Once seen, my method of artificial respiration is instantly understood, easily remembered, and readily practiced, by any person of ordinary intelligence.

Leroy, whose experiments antedate those of Marshall Hall, enforced expiration by compressing the chest with bandages, then, relaxing these, he trusted to the resiliency of the costal cartilages to expand the chest, and so to secure inspiration He appears to have taken no care to guard against obstruction of the glottis by the tongue. So early as 1829, he affected artificial respiration by applying the interrupted galvanic current directly to the diaphragm. His efforts were praiseworthy, and not unfruitful; for, besides stimulating the investigations of Marshall Hall, they led Dr. Charles Kidd, in 1865, to Faradization of the phrenic nerve and diaphragm, whereby he resuscitated a woman apparently dead from chloroform.

There have been various plans for inflating the lungs by means of bellows and tube. But they are not without danger of injury to the lungs, and the apparatus cannot always be at hand. Even when present, Dr. Marcet, one of their advocates, advises "to begin with the Ready Method, in order to lose no time." M. Marchant proposes to insert a tube, like a tobacco pipe, in one nostril, closing the other nostril and the mouth, and blowing through the tube. This process, which can succeed only in case the glottis be unobstructed, is not new. It was, in 1817, minutely described in Hosack's edition of "Thomas' Practice," page 729. Allusion is also there made to Hunter's bellows, "which is of such construction, that by one action, fresh air is thrown into the lungs, and by another it is thrown out again, so as to imitate, or produce artificial breathing." Would not Dr. Dewees have found, in the suction power of Hunter's bellows, the desideratum for his new-born patients, when, in 1826, he says, "Might not a properly constructed syringe be highly useful in removing (from the trachea) the obstructing mucus?" What solid pleasure good Dr. Dewees must have taken in manipulating his little pets, when placing the child's mouth downward, and holding the body and hips higher than the head; at the same time gently shaking the child, so as to disengage any mucus that may be lodged in the trachea, and permitting it to flow from the mouth; then patiently inflating the lungs of the child, by forcing air into them from his own, and next expelling it, by pretty firm pressure on the thorax, by which means he believed that he had saved the lives of many children.

Dr. Dewees knew the fact, but not the reason, of the injurious effect of the warm bath during the interruption of all respiration. Some interesting experiments made by Dr. B. W. Richardson, of London, and others; by Dr. Joseph G. Richardson, of Union Springs, N. Y., attest the value of hot air, internally and externally, when applied at the same time as artificial respiration.

Dr. C. Handfield Jones, in March, 1869, details a postural mode of inducing artificial respiration in asphyxiated children. He says, "I laid him down on his back, and made pressure on his abdomen; then raised him upright on his seat, and so on alternately."

Dr. Billman, of Neustadt, writing in 1867, eulogises catheterization of the trachea and suction, not only for the removal of obstructing mucus and foreign bodies, but as a means of resuscitation in all forms of suspended animation. In the asphyxia of new born children, Dr. Lowenhardt uses "a pump and a fine india rubber tube, ten inches long, with catheter openings at the end. This tube is inserted by the aid of a fine stilet into the trachea, in the following way: "An assistant, with thumb and finger, presses the neck above the larynx, closing the œsophagus, whilst the operator depresses the tongue with his forefinger, and slips in the tube. This tube is then attached to the aspirating, pump which is used to draw out the obstructing fluids; then air is gently introduced."

Is not this process adapted to some cases of croup, diphtheria and scarlatina? I think it is; at Elmira. in 1842, I rescued from inevitable death a boy of twelve years, by passing curved forceps into the larynx and drawing thence a mass of stringy mucus. This was in a case of scarlatina. The patient was a son of the late Mr. Hammond Sly, and I believe that he is still living.

As a further means of resuscitation, Dr. B. W. Richardson has well said, "The great desideratum now is an improved method of artificial circulation of the blood."—*Med. & Surg. Reporter.*

---

